# Proposal for Computationally Efficient Fog Computing System for Coffee Berry Borer Detection via Optimized YOLOv26

**DOI:** 10.3390/s26072212

**Published:** 2026-04-03

**Authors:** Ingrid P. Huaman-Pacco, Erwin J. Sacoto-Cabrera, Vinie Lee Silva-Alvarado, Ali Ahmad, Sandra Sendra, Jaime Lloret, Edison Moreno-Cardenas

**Affiliations:** 1 TESLA Laboratory, Universidad Nacional de San Antonio Abad del Cusco, Cusco 08003, Peru; 182972@unsaac.edu.pe; 2 GIHP4C, Universidad Politécnica Salesiana, Cuenca 010102, Ecuador; esacoto@ups.edu.ec; 3 Instituto de Investigación para la Gestión Integrada de Zonas Costeras, Universitat Politécnica de València, C/Paranimf, 1, 46730 Grao de Gandia, Valencia, Spain; vlsilalv@doctor.upv.es (V.L.S.-A.); aahmad1@upv.es (A.A.); sansenco@upv.es (S.S.); jlloret@dcom.upv.es (J.L.); 4 Technology and Engineering Group, EM Research & Tech, Cusco 08003, Peru

**Keywords:** object optimization, precision agriculture, advanced AI, CNN, image analysis, machine learning, WSN, fog computing

## Abstract

**Highlights:**

This research develops a computationally efficient system for real-time pest monitoring in complex agricultural environments. The key findings are:

**What are the main findings?**

- FasterNet and SimSPPF reduced model parameters while enhancing mAP@0.5.
- Pareto analysis identified the optimal balance between detection accuracy and computational overhead.

**What are the implications of the main findings?**

- Proposal for a structured optimization allows high-fidelity pest monitoring on low-power for fog devices.
- Structural efficiency can be prioritized without sacrificing detection reliability.

**Abstract:**

The Coffee Berry Borer is the most destructive pest affecting global production of *Coffea arabica*. Early detection of pest-induced fruit damage remains challenging due to the small size of infestation symptoms and the dense clustering of coffee berries under complex field conditions. This study evaluates optimized object detection architectures designed to improve the balance between detection accuracy and computational efficiency. Three baselines were established: YOLOv8n (M0), YOLOv11n (M1), and YOLOv26n (M2). Seven architectural variants (M3–M9) were then developed by integrating FasterNet, SimSPPF, and EMA. Experimental results showed that M0 achieved the highest detection accuracy (mAP@0.5 = 0.9534 and 6.09 GFLOPs), whereas model M6, combining FasterNet and SimSPPF, provided the best accuracy–efficiency trade off with mAP@0.5 = 0.9446 and 5.12 GFLOPs. Pareto analysis confirmed M6 as the optimal configuration. Finally, in situ validation across 25 points achieved a mean F1-score of 0.7255 (SD = 0.0504) for infected berries despite cast shadows, proving its readiness for real-time agricultural deployment.

## 1. Introduction

The Coffee Berry Borer (CBB), scientifically known as *Hypothenemus hampei*, is universally recognized as the most destructive pest affecting the cultivation of Coffea arabica, severely compromising both yield and final bean quality [1]. At the global scale, the economic damage caused by *H. hampei* is substantial, with estimated annual losses exceeding USD 500 million, a figure that can increase markedly under high infestation scenarios [2]. In addition to direct damage, recent studies demonstrate a strong association between insect perforation and the increased incidence of coffee fruit rot (CFR), since the pest can act as a vector of pathogenic fungi such as *Colletotrichum* and *Fusarium* [1]. Effective management remains particularly challenging because the insect completes most of its life cycle inside the coffee berry, thereby limiting the effectiveness of external control measures to very narrow time windows during the infestation process [3].

This pressure is especially pronounced in high value coffee producing regions [4], where attack rates in conventional plantations may range from 28% to 41% of the crop [5]. Consequently, reliance on traditional control approaches has become increasingly unsustainable. Historically, pest management strategies against *H. hampei* have relied heavily on chemical pesticides, which have demonstrated considerable drawbacks, including pesticide residues in coffee products, the emergence of pest resistance, and adverse impacts on both human and environmental health [6]. Moreover, the financial burden associated with these agrochemicals often renders them economically inaccessible for the majority of smallholder farmers who dominate global coffee production systems [2].

Within this context, the integration of artificial intelligence (AI) and computer vision has emerged as a transformative strategy for advancing precision agriculture (PA) [7], enabling the early identification of phytosanitary threats with high accuracy [8]. In contrast with conventional monitoring approaches, deep learning (DL) models, particularly convolutional neural networks (CNN), provide powerful tools for rapid and precise visual recognition tasks in agricultural environments [9]. Such capabilities directly support integrated pest management (IPM) by optimizing resource allocation and enabling crop protection treatments to be applied exclusively in affected areas [10]. Furthermore, the need for real-time and localized visual data processing has accelerated the adoption of edge artificial intelligence (Edge AI) solutions in agricultural systems [11]. Deployment of DL models on low-cost edge computing platforms, including devices such as the Raspberry Pi, represents a critical step toward enabling automated crop monitoring in rural environments, ensuring mobility, reduced latency, and broad technological accessibility [12].

Despite the rapid expansion of DL applications in agriculture, an important technical gap persists in the detection of subtle pest damage in densely clustered fruits, particularly when employing state-of-the-art ultralight architectures [9]. Detection of *H. hampei* damage represents a particularly demanding computer vision (CV) problem due to the extremely small and often occluded nature of the infestation signs within complex coffee clusters [2,5,9,13]. Accurate identification of these early stage perforations, therefore, requires highly robust object detection strategies capable of distinguishing subtle visual patterns within heterogeneous and cluttered scenes [8,14].

Addressing this challenge requires the development of efficient, scalable, and field- deployable monitoring technologies capable of operating under real agricultural conditions. Accordingly, this study proposes an advanced artificial vision framework designed for practical phytosanitary monitoring of coffee crops. The research specifically focuses on three key objectives. First, implementation and optimization of an object detection system based on the YOLOv26n architecture are performed to enable rapid detection of CBB- related damage. Second, an empirical validation of a bounding box annotation strategy is conducted using a mixed dataset characterized by high visual complexity. Third, system performance is quantified in terms of detection efficiency and computational latency when deployed on a low-cost fog computing platform under real-world conditions. The computational metrics of the fog computing system were not evaluated in this study since this study lays the foundation for the selection of best CV model and a thorough evaluation of the proposed fog system would be necessary.

The remainder of this paper is organized as follows. Section 2 reviews the most relevant studies reported in the literature. Section 3 presents the materials and methods employed in the experimental framework. Section 4 reports the results obtained with a comprehensive discussion. Finally, Section 5 summarizes the main conclusions and outlines future research directions.

## 2. Related Work

Computer vision has emerged as a transformative technological paradigm in PA, providing advanced tools for the timely and accurate identification of plant diseases and pest infestations. Within the context of coffee crop monitoring, several studies have demonstrated the potential of DL approaches to enhance phytosanitary diagnostics. For instance, Adelaja and Pranggono [15] conducted a comparative investigation on coffee leaf disease detection using CNN. Their study showed that advanced architectures such as VGG16 can achieve classification accuracies of up to 94.60% when trained on multiple benchmark datasets, while the specialized model CoffNet achieved a processing speed of 125.93 frames per second. These findings demonstrate the capacity of CNN-based systems to deliver both high accuracy and rapid inference in plant pathology applications.

Following this direction, Gomez et al. [16] extended the application of DL techniques to the detection of major diseases affecting *Phaseolus vulgaris*. Their framework employed a large-scale dataset containing more than 9500 images and approximately 44,000 expert annotations. The implementation of the YOLO–NAS architecture achieved a mean average precision (mAP) of 97.9% and a recall of 98.8% in the detection of leaf pathologies. Similarly, in the domain of agricultural product inspection, Wang et al. [17] proposed WT-YOLOM, an enhanced DL algorithm designed for the detection of difficult endogenous impurities in walnuts. The optimized model, derived from YOLOv4, achieved an average precision of 94.4%, while maintaining a processing speed of 60.1 frames per second and reducing the model size by 88.6% relative to the baseline architecture. Collectively, these studies highlight the growing effectiveness of YOLO-based object detection models for complex agricultural inspection tasks.

Despite these advances, the detection of the CBB remains particularly challenging because infestations occur within densely clustered coffee fruits, frequently under conditions of occlusion and visually complex backgrounds. Such characteristics complicate object localization and demand highly optimized architectures capable of detecting small and partially hidden targets. Addressing these challenges, Ye et al. [8] developed a lightweight one-stage CNN model based on YOLOv7 to identify coffee fruit maturity. Their architecture introduced the L Norm Attention mechanism together with SPD Conv to improve the detection of small and occluded objects in low resolution imagery. Experimental results achieved an mAP@0.5 of 80.1% and a recall of 78.4% in scenarios characterized by dense fruit distribution and partial occlusion, demonstrating the importance of attention mechanisms in visually complex agricultural environments.

In parallel, the pursuit of efficient lightweight detection models has stimulated the development of architectures specifically designed for rapid pest identification. For example, Zheng et al. [18] proposed Rice YOLO, a detection framework derived from YOLOv8 for the rapid identification of rice pests under conditions of high visual similarity and complex occlusion. The improved architecture incorporated an efficient detection head, deep supervision strategies, and a dynamic oversampling module. Experimental evaluation demonstrated that Rice YOLO outperformed several baseline models, achieving an mAP@0.5 of 78.1% and an mAP@0.5:0.95 of 62.9%.

Practical deployment requires precise localization and efficiency for field conditions, shifting research toward parameter reduction and edge-compatible architectures. In this context, Gong et al. [14] optimized YOLOv7-tiny for resource-constrained environments using a lightweight backbone and CotNet Transformer. This approach achieved a 92.8% mAP@0.5 with a 4.0 ms inference time, outperforming Faster R-CNN and SSD in both speed and accuracy. Further emphasizing field deployment capabilities, Zhang et al. [19] proposed GVC YOLO, a lightweight architecture derived from YOLOv8n for the edge-based monitoring of aphid damage on cotton leaves. The model prioritized localization accuracy and operational efficiency, achieving an mAP@0.5 of 97.9% while maintaining a compact size of only 5.4 MB. When optimized using TensorRT on the Jetson Xavier NX platform, the system reached a processing speed of 48 frames per second, demonstrating suitability for large-scale agricultural monitoring applications. Complementarily, Li et al. [20] proposed YOLOv11 GS, a framework derived from YOLOv11n with oriented bounding boxes for the precise localization of harvesting points in automated grape harvesting systems. The architecture integrated BiFPN and Ghost modules to reduce model parameters by 28.3%, achieving an mAP of 89.5% and a positioning error of only 8.63 pixels, thereby demonstrating strong potential for deployment on mobile platforms.

Although the existing literature confirms the effectiveness of YOLO-based architectures for agricultural pest detection, most reported studies primarily focus on leaf classification tasks or object detection under relatively less complex visual conditions. Empirical validation of the state-of-the-art YOLOv26n architecture for the detection of subtle damage caused by *H. hampei* in clustered fruits of Coffea arabica remains largely unexplored. In particular, limited research has examined the integration of robust annotation strategies with lightweight DL models deployed on low-cost edge computing platforms capable of real-time operation in field environments. This research addresses this critical gap by evaluating an optimized detection framework capable of identifying early CBB damage with possible high precision and likely ultralow latency when deployed on the Raspberry Pi 5 platform. The proposed approach therefore contributes to a practical and scalable technological solution for phytosanitary monitoring of a pest with major global economic impact.

## 3. Materials and Methods

**Figure 1:**
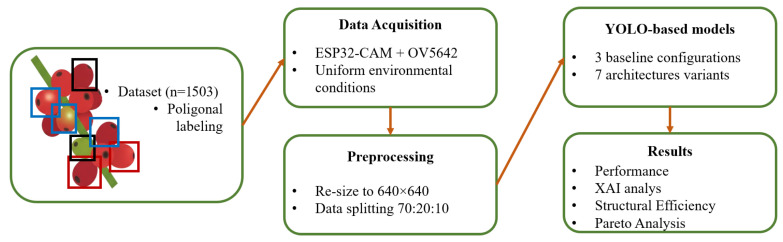
Methodological flowchart of the proposed coffee monitoring system, from image acquisition and dataset preparation to model optimization and performance evaluation.

The methodological framework for this study is synthesized in Figure 1, detailing the progression from data acquisition using ESP32-CAM sensors (Ai-Thinker, Shenzhen, China) to the final Pareto analysis. This section describes the dataset preparation, the integration of custom YOLO-based architectures, and the evaluation metrics used to validate the fog computing system.

### 3.1. Coffee Berry Borer Dataset

The images used in this study were collected from coffee cultivation plots located in Chaullay, Cusco, Peru. Image acquisition was performed using an ESP32-CAM module equipped with an OV5642 sensor, featuring a 5-megapixel resolution. This specific hardware configuration was chosen to ensure that the training dataset directly reflects the optical characteristics and constraints of the sensors deployed in the final Wireless Sensor Network (WSN).

The dataset was specifically designed to support the development of an automated monitoring system for detecting coffee berries and identifying symptoms caused by the CBB (*Hypothenemus hampei*). In order to ensure data consistency, all images were captured under the uniform environmental conditions across multiple days. Manual annotation was conducted using bounding boxes to label three primary classes: healthy, damaged, and infected coffee berries, as well as berry clusters. The resulting dataset consists of 1503 annotated images, partitioned into 70% for training, 20% for validation, and 10% for testing with polygonal labelling. In order to evaluate model performance under diverse conditions, experiments were conducted using different YOLO architectures. Prior to the training phase, all images were pre-processed by resizing them to 640 × 640 pixels to standardize the input format for the convolutional neural network (CNN). The dataset incorporates significant variability in fruit maturity, spatial distribution, and light conditions, which improves the robustness and generalization capability of the models.

### 3.2. Coffee Monitoring Using Wireless Sensor Network

**Figure 2:**
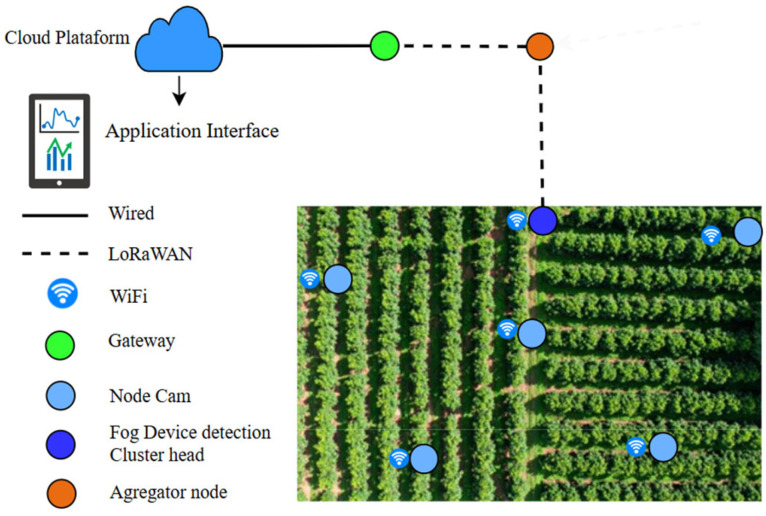
Hierarchical architecture of the Wireless Sensor Network (WSN) for coffee monitoring, integrating Fog Computing multi-protocol connectivity.

The proposed monitoring system is structured as a multi-tier Fog Computing architecture designed to achieve 100% spatial coverage across extensive coffee plantations while maintaining strict bandwidth and energy efficiency (Figure 2). This hierarchy optimizes task distribution and network load balancing [21]. At the ground level, the network is composed of Node Cams (ESP32-CAM with OV5642 sensors) organized into dynamic sub-clusters. These nodes act as lightweight edge sensors whose primary function is the acquisition and transmission of raw visual data.

A critical challenge in these environments is the signal attenuation caused by dense foliage. Following the radio design principles for rural areas established by Lloret et al. [22], the received power ($P_{rx}$) is significantly affected by vegetation loss ($L_{vegetation}$), with a recommended attenuation factor of 1.2 dB/m for 802.11 g networks in forested or agricultural zones. In coffee plots, where the leafiness can be high, this limits the reliable WiFi coverage distance between the Node Cam and the Cluster Head to approximately 30–34 m when significant vegetation intercedes in the line of sight.

The Cluster Head (CH), acting as the Fog Device (Raspberry Pi 5 + AI HAT+), executes the optimized YOLOv26 model on the images captured by the Node Cams. When a Coffee Berry Borer infestation is detected, the CH generates a lightweight metadata packet. This information is transmitted via LoRaWAN to an Aggregator Node, which forwards the data to a LoRa Gateway. The gateway, connected via a wired link, syncs the detection alerts to the Cloud Server. This low-power pathway ensures that critical phytosanitary alerts are delivered even in areas with poor WiFi coverage.

**Figure 3:**
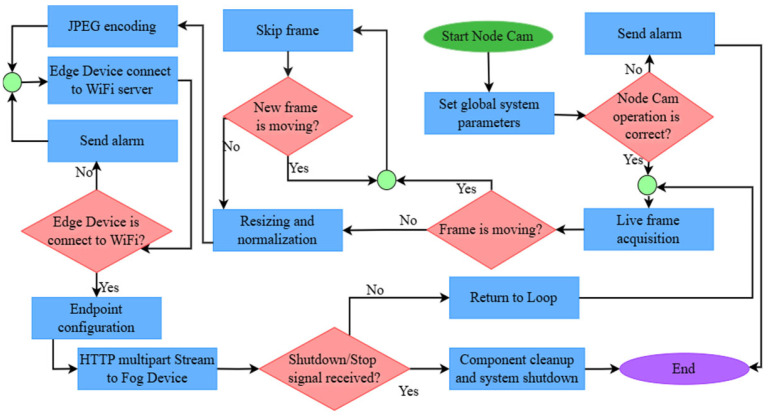
Operational flowchart of the Node Cam for real-time coffee monitoring. This node represents one of the five identical units within a localized cluster, designed for synchronized data acquisition and streaming to a central Fog Device.

**Figure 4:**
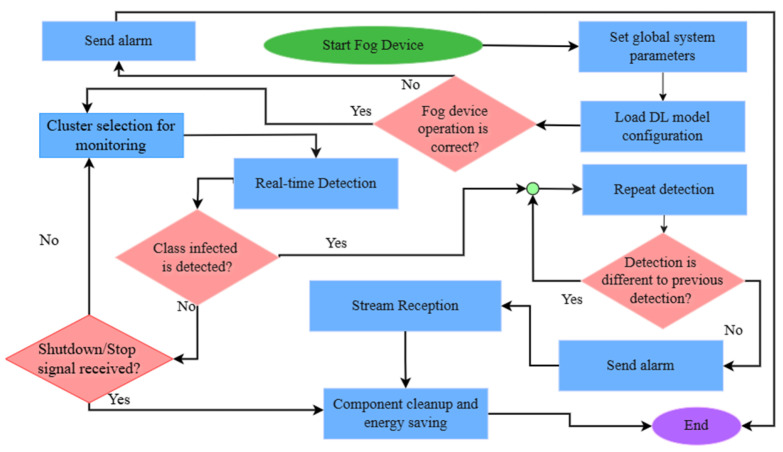
Operational flowchart of the Fog Device for automated detection and real-time coffee detection.

The system operates under a Remote Inference Protocol to minimize energy consumption and bandwidth usage. In this hierarchy, the Node Cam (ESP32-CAM) follows a scheduled polling mechanism orchestrated by the Fog Device (Cluster Head) (Figure 3 and Figure 4). Instead of continuous data transmission, each node performs initial frame filtering and establishes an HTTP Multipart Stream only during its assigned temporal slot or upon receiving a specific trigger from the Fog Device. This ensures that high-bandwidth visual data is transmitted for processing only when the node is active, effectively preventing spectral congestion in the 2.4 GHz band. The core computational workload is offloaded to the Cluster Head, which functions as the primary Fog Computing unit. Leveraging its 40 TOPS hardware acceleration (Raspberry Pi 5 + AI HAT+), the CH executes the optimized YOLOv26 model to identify the Coffee Berry Borer (CBB) in real-time. This centralized processing allows the system to transition to Point-to-Point Mode. This real-time control loop ensures possibly low-latency data distribution [23].

### 3.3. YOLO-Based Architectures

The YOLO architectures are characterized by a balance between computational latency and mAP. Currently, YOLOv26 stands as a benchmark for real-time object detection due to its optimized feature extraction layers. However, while the vanilla YOLOv26 architecture excels in general-purpose benchmarks, its deployment in resource-constrained environments—such as coffee plantations—requires further refinements to manage energy consumption and complex background noise.

**Figure 5:**
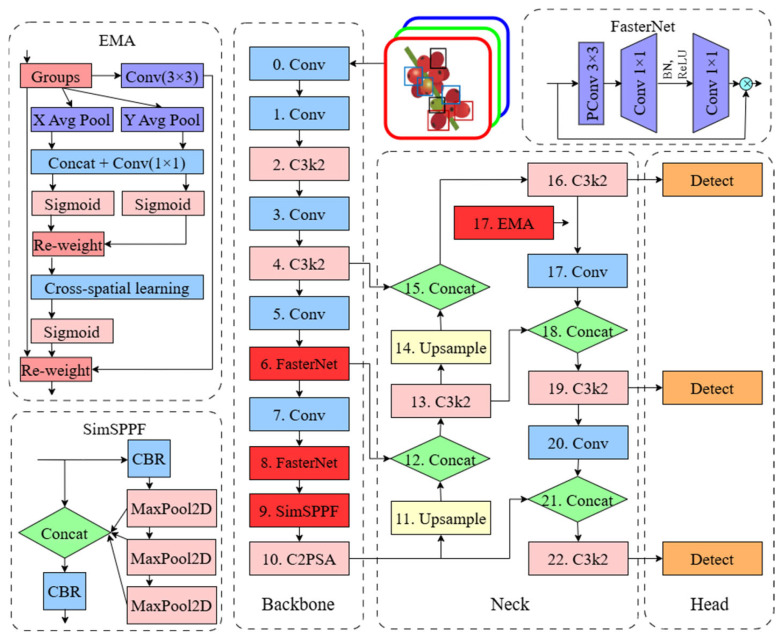
Detailed architecture of the proposed YOLOv26-based model, integrating Faster Block, SimSPPF, and EMA modules for optimized coffee berry detection.

In order to address the computational limitations of standard convolutions, structural modifications were implemented. In the model’s backbone, FasterNet blocks replaced the C3k2 layers at stages 6 and 8. These blocks utilize Partial Convolutions (PConv), which restrict the convolution operation to a subset of input channels while treating the remainder as an identity mapping. As shown in Figure 5, this mechanism reduces computational redundancy and memory bandwidth without compromising the effective receptive field [24]. Another theme of interest is that the detection of coffee berries is hindered by high variability in fruit size and spatial overlap within clusters. The Simple Spatial Pyramid Pooling–Fast (SimSPPF) module was incorporated to enhance multi-scale feature extraction. This module replaced the standard SPPF at layer 9. This layer performs parallel pooling operations to capture both local and global context. Unlike standard SPPF, SimSPPF utilizes a simplified pooling structure that preserves spatial information for small-object detection while maintaining low latency in the processing pipeline [25]. Furthermore, to improve feature discrimination under inconsistent lighting and high occlusion, the Efficient Multi-Scale Attention (EMA) module was integrated. Unlike previous modifications, EMA was inserted as a new functional layer at position 17, shifting the index of subsequent layers. EMA re-weights spatial and channel-wise features through a multi-scale approach that retains cross-dimensional information without a linear increase in parameters [26]. This specific placement allows the network to prioritize visual cues of *Hypothenemus hampei* infestation, such as small entry perforations, against complex foliage backgrounds.

### 3.4. Training and Validation Protocol

**Figure 6:**
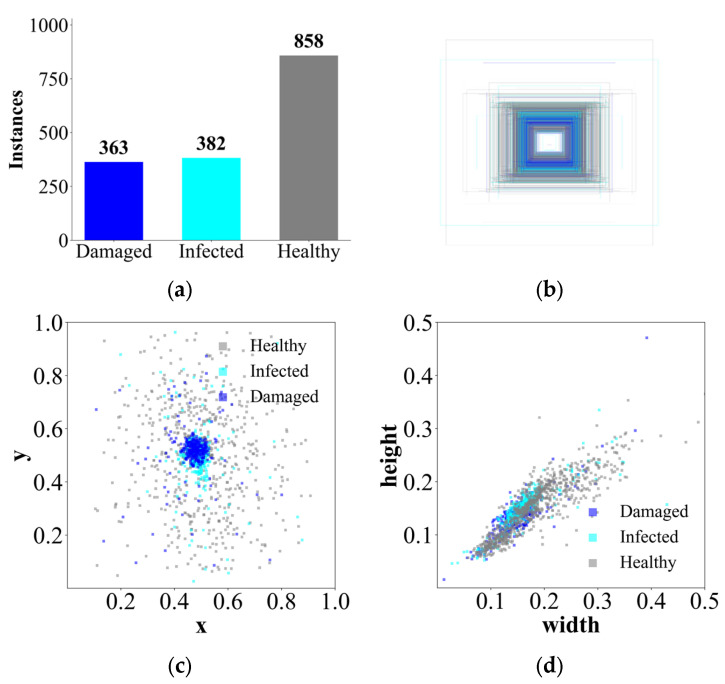
Structural overview of the annotated labels: (**a**) Frequency of samples per category. (**b**) Superimposed bounding box coverage within the image frame. (**c**) Spatial distribution of centroid locations in normalized coordinates. (**d**) Morphological distribution analyzing the correlation between bounding box width and height.

The dataset consists of 1503 annotated images, partitioned into 70% for training, 20% for validation, and 10% for testing (Figure 6) as reported previously [27,28]. In order to ensure consistency and a fair comparison across the hierarchical monitoring system, all models were trained using a deterministic approach with a fixed random seed, synchronizing all hyperparameters to eliminate stochastic fluctuations between runs. The training process was conducted for a total of 100 epochs with a batch size of 16 and a fixed input resolution of 640 × 640 pixels. A differentiated initialization strategy was implemented, while the baseline models (M0, M1, and M2) utilized a transfer learning strategy initialized with COCO pre-trained weights [29,30], the proposed architectural variants (M3 through M9) were trained from scratch. This ensures that the performance gains observed in the optimized models are strictly attributable to the structural integration of FasterNet, SimSPPF, and EMA modules, rather than inherited feature representations.

During the validation phase, a minimum confidence threshold of 0.001 was utilized to construct the Precision–Recall curves and calculate the mAP metrics accurately. The performance evaluation was conducted on both the validation and testing datasets, utilizing DL metrics standardized in the literature [8,11,17,19,31,32,33], including Precision, Recall, F1-score, Accuracy, mAP@0.5, and mAP@0.5:0.95. However, for the real-time field deployment on Raspberry Pi 5, a confidence threshold of 0.50 was established for the inference pipeline. This threshold was selected to balance the sensitivity required to detect subtle borer entry holes with the need to minimize false alarms caused by environmental noise or surface irregularities on the coffee berries.

### 3.5. Multi-Objective Model Selection

In order to identify the optimal architecture for edge deployment, a multi-objective evaluation framework was developed. The performance of each variant was quantified using three specialized indices designed to measure the trade-offs between diagnostic accuracy and hardware resource consumption. Specifically, the Latency Performance Score (LPS) was formulated to evaluate the precision yield per millisecond of inference, serving as the primary indicator for real-time viability. The GFLOPs Efficiency Score Index (GESI) quantifies the algorithmic by relating accuracy to the theoretical computational load. Finally, the Parameter Efficiency Index (PEI) measures the model’s capacity to maximize detection performance relative to its total parameter count. In order to provide a unified ranking, a Custom Score (S) was implemented as an equitable weighted average of these normalized metrics. This equal-weighting scheme (25% per metric) ensures that the selection is not biased toward a single performance dimension, but rather identifies the architecture that provides the most stable trade-off between predictive power for all classes (F1-score), inference speed (LPS), parameter utilization (PEI), and energy-computational efficiency (GESI). This approach is critical for agricultural edge nodes where hardware constraints are as significant as diagnostic accuracy. Furthermore, a Pareto Frontier Analysis was applied to identify non-dominated models, ensuring that the selected architecture offers the best possible balance between accuracy and complexity. The mathematical formulation for these criteria are detailed in Table 1.

## 4. Results and Discussion

This section presents a comprehensive evaluation of the different architectures, analyzing their performance across multiple operational dimensions. The discussion is structured to provide a holistic view of the capabilities of models, first by examining the Convergence Analysis during the training phase, followed by a Statistical Evaluation of diagnostic accuracy and Structural Efficiency through Pareto optimization. Furthermore, the model’s reliability is validated via Explainable Artificial Intelligence (XAI) to ensure transparency in decision-making. Finally, the findings are contextualized through a Comparative Analysis with the current literature, concluding with an objective review of the Limitations and Future Perspectives for its deployment in autonomous coffee crop monitoring.

### 4.1. Convergence Analysis

**Figure 7:**
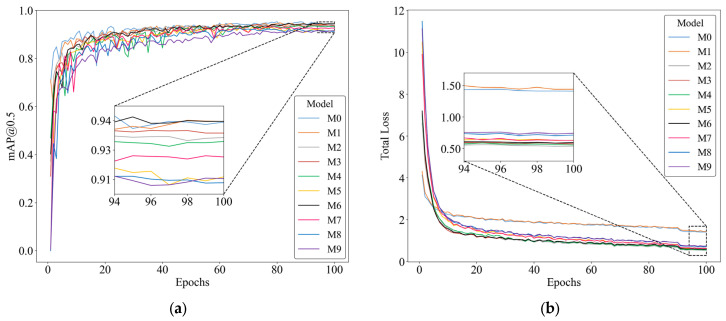
Training performance evaluation for the proposed YOLO models. (**a**) Mean Average Precision (mAP@0.5) performance progress. (**b**) Total loss evolution over 100 epochs. Insets provide a detailed view of convergence during the final six epochs.

The learning behavior and stability of the ten evaluated YOLO variants (M0–M9) were monitored over 100 epochs to ensure convergence and assess architectural robustness. The progression of the mAP@0.5 metric, illustrated in Figure 7a, serves as the primary indicator of detection accuracy. While all configurations reach a precision plateau beyond the 60th epoch, the structural modifications lead to distinct performance levels. By the 100th epoch, the proposed M6 variant achieves the highest detection accuracy with an mAP@0.5 of 0.94467, slightly outperforming both the baseline M0 (0.94424) and the M1 variant (0.94463). This peak precision is particularly significant as M6 maintains reduced computational complexity, confirming that the architectural enhancements effectively capture the features of the Coffee Berry Borer (CBB).

Complementarily, Figure 7b illustrates the total loss evolution, which exhibits a sharp exponential decay during the first 20 epochs across all models. A steady stabilization is observed from the 94th epoch onwards, indicating that the network weights have reached a reliable state for inference. Notably, the baseline models (M0 and M1) maintain significantly higher final loss values (above 1.41) compared to the modified versions. Models M2, M3, M4, and the proposed M6 demonstrate superior optimization efficiency, with final loss values ranging between 0.53 and 0.59. This reduction of more than 50% in the error magnitude relative to the baseline suggests that the integration of FasterNet and SimSPPF modules facilitates a more precise convergence of the objective function.

### 4.2. Statistical Evaluation of Model Performance

The experimental results of the test for all architectural variants are summarized in Table 2. The models were evaluated based on mAP, Precision, Recall and computational efficiency such a Parameters and GFLOPs.

The comparative analysis of the proposed architectures, grounded in the results obtained from the test dataset, reveals a significant evolution in structural efficiency from the reference models toward the YOLOv26 variants. By establishing M1 (YOLOv11n) as the state-of-the-art baseline, it is observed that the M2 base configuration achieves an 8% reduction in parameter count and a 17.7% optimization in GFLOPs while maintaining a mAP@0.5 of 0.9426. This marginal loss of only 0.4 percentage points compared to the baseline validates the architectural simplification strategy for edge computing environments.

The ablation study identifies the individual and combined impact of the integrated modules. The adoption of the FasterNet in model M3 reduces computational complexity to its minimum level at 5.124 GFLOPs, demonstrating the superiority of partial convolutions in managing data flow with reduced redundancy. Furthermore, the architectural optimization of the M6 variant is characterized by a strategic replacement of standard components to enhance the accuracy-to-efficiency ratio. Specifically, the integration of FasterNet blocks to replace the conventional C3k2 modules resulted in a 2.37% reduction in total parameters, a 1.31% decrease in GFLOPs and a 0.21% increase in mAP@0.5 compared to the YOLOv26n baseline (M2). Furthermore, the transition from standard SPPF to SimSPPF was implemented to prioritize inference speed; while this modification maintains an identical parameter count, it simplifies the mathematical operations within the spatial pooling layer.

In contrast, the incorporation of the EMA attention mechanism in variants M5, M7, M8, and M9 shows a downward trend in precision and recall metrics, with mAP@0.5 dropping to 0.9142 in the densest configuration (M9). This phenomenon suggests that in nano-scale models intended for coffee pathology detection, the saturation of attention modules may introduce unnecessary complexity that hinders convergence compared to the direct efficiency of simplified spatial blocks.

Furthermore, it is essential to highlight that while the M0, M1, and M2 models benefited from fully pre-trained weights, the modified architectures (M3–M9) were trained from scratch (from-scratch training). The fact that models like M6 achieved an mAP@0.5 of 0.9446—nearly matching the pre-trained state-of-the-art—without the advantage of prior knowledge, suggests a significant margin for improvement. This implies that the proposed structural modifications are inherently robust, and their performance could be further amplified through large-scale pre-training or transfer learning, potentially surpassing the current baselines in both accuracy and efficiency.

### 4.3. Structural Efficiency and Pareto Analysis

**Figure 8:**
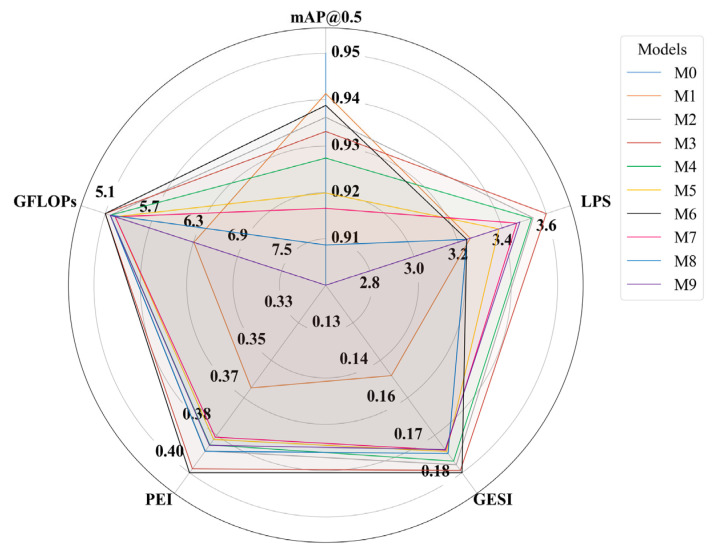
Multi-objective performance profiling of YOLO variants (M0–M9). In this representation, the GFLOPs axis has been inverted; therefore, values closer to the periphery indicate lower computational demand and, consequently, higher operational efficiency.

In order to determine the viability of the models in edge computing environments, a multi-objective analysis was performed, represented by the radar profiling chart in Figure 8. This visualization integrates five critical dimensions: mAP@0.5, LPS, GESI, PEI, and GFLOPs.

The visualization identifies the M0 model (light blue) which defines the upper limit of precision with an mAP@0.5 of 0.9534. However, this performance comes at the cost of significantly reduced structural and energy efficiency. Visually, the M0 profile collapses into a nearly vertical line toward the mAP@0.5 vertex, while dropping to the minimum values in all other categories. On the other hand, the M2, M3, and M4 models show outstanding performance on the LPS axis, with values of 3.566, 3.627, and 3.559, respectively, positioning them as the fastest candidates for real-time inference. In contrast, the proposed M6 model emerges as the optimal solution under a Custom Score criterion that balances lightness and speed without drastically sacrificing detection capability. The M6 model successfully expands its area toward the efficiency vertices, achieving a PEI of 0.4073 and a GESI of 0.1843, even surpassing all models in terms of sustainability and parameter utilization. The M6 model maintains a competitive accuracy of 0.9446—only 0.0088 points below the M0—while operating with a load of only 5.124 GFLOPs, matching the computational efficiency of the lightest model with M3.

The quantitative support for this selection is provided in Table 3, which ranks the models based on their Custom Score and identifies those belonging to the Pareto Frontier. Although M0 and M1 are technically on the frontier due to their mAP@0.5, M6 achieves the highest balanced performance. As shown in Table 1, M6 is identified as the superior architecture (Yes in Pareto Frontier), validating that the strategic integration of efficiency-focused modules provides the best trade-off for resource-constrained deployment.

Finally, it is observed that the saturation of attention modules in the M7, M8, and M9 variants causes a degradation in the precision axis, shifting their profiles toward the center of the radar. Specifically, in the M9 model, the increase in structural density does not translate into performance gains, resulting in the lowest mAP@0.5 of the comparison.

Following the comparative evaluations, M6 was selected as the optimal architecture to undergo an in situ field performance analysis as reported previously [34].

### 4.4. In Situ Field Performance Analysis Results

**Figure 9:**
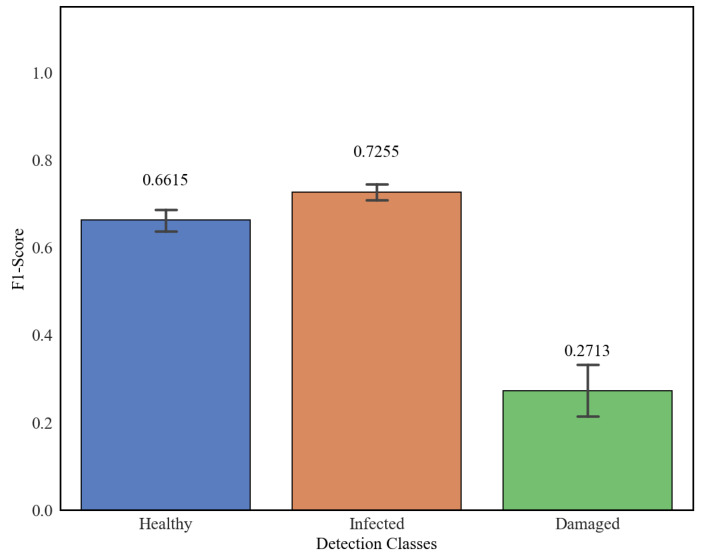
Mean F1-score and statistical dispersion for Healthy, Infected, and Damaged classes during in situ validation of the proposed M6 model. Error bars represent the 95% confidence interval (n = 25) at a confidence threshold of 0.25.

In order to evaluate the operational reliability of the proposed M6 model, an in situ experimental validation was conducted across 25 independent monitoring points (*n* = 25). The sensor nodes maintained a standardized distance of 50 cm from the coffee berry clusters. For all field tests, the Confidence Threshold was set to 0.25. The results of this evaluation, which are visually summarized in Figure 9, reflect the system’s performance under real-world agricultural conditions characterized by uncontrolled lighting and complex canopy architectures.

Regarding the primary target of this study, the Infected class demonstrated the highest level of accuracy and stability. The model achieved a mean F1-score of 0.7255, with a remarkably low standard deviation (SD = 0.0504). The high precision of this estimate is evidenced by a narrow 95% confidence interval (0.7047–0.7463). While individual measurements reached a maximum of 0.8147 under optimal visibility, the performance maintained a floor of 0.6492, even in challenging locations.

**Figure 10:**
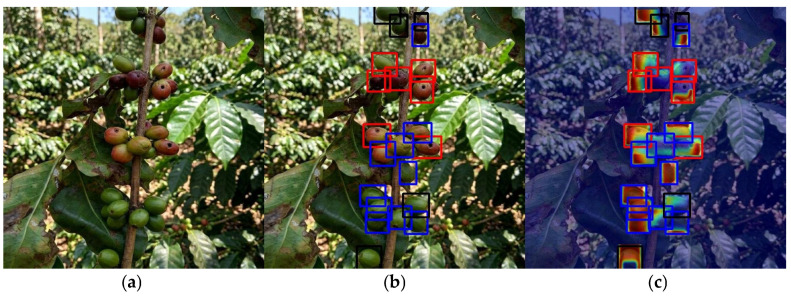
XAI-based interpretability analysis of coffee berry pathology detection using Eigen-CAM for M6. The figure illustrates the model’s decision-making process through three panels: (**a**) Original field image. (**b**) Object detection results with color-coded bounding boxes: Healthy (Blue), Infected (Red), and Damaged (Black). (**c**) Eigen-CAM heatmaps demonstrating the spatial correlation between high-activation zones and the specific morphological features of the berries. The quantitative performance for this specific field sample is an F1-score of 0.7143 for the Infected class and 0.7273 for Healthy berries, while identifying the Damaged class as 0.2857.

The Healthy class also showed reliable performance with a mean F1-score of 0.6615. However, this category exhibited a wider range of values, with a maximum of 0.7547 and a minimum of 0.5576 (SD = 0.0639). These lower values (Min: 0.5576) are primarily attributed to false-positive misclassifications where the model confused healthy berries with the Damaged class (Figure 10). This phenomenon occurs because cast shadows from the coffee leaves create irregular dark patterns on the berry surface. Since the model associates non-circular dark patches with necrotic tissue, it often misidentifies these shaded healthy berries as Damaged, in accordance with the results reported previously [8].

In contrast, the Damaged class presented the most significant challenges, resulting in a mean F1-score of 0.2713 and the highest degree of uncertainty (SD = 0.1569). While some points reached a maximum of 0.5020, others fell to a critical minimum of 0.0493. This low performance floor is directly linked to environmental noise. Specifically, when the characteristic entry holes of an active infection (Infected) were located precisely within shaded zones, the geometric features of the perforation were masked by the low-reflectance signature of the shadow. In such cases, a small percentage of Infected instances were erroneously labeled as Damaged or omitted entirely, similar findings have been reported previously [28].

### 4.5. XAI Analysis

In order to validate the reliability of the M6 model’s decisions and ensure that the detection of coffee fruit pathologies is not the result of spurious correlations, an XAI analysis was implemented using the Eigen-CAM algorithm. This technique visualizes the hierarchical importance of learned features by projecting activation maps onto the final layers. The analyzed samples were obtained directly from the in situ field video stream. As observed in Figure 10, there is a precise spatial correlation between the hotspots of high semantic activation and the regions manifesting symptoms of Healthy (Blue), Infected (Red), and Damaged (Black).

Notably, the model maintains high discernment capacity even in critical scenarios of partial occlusion, where fruits are partially covered by foliage, branches, or overlapping clusters. The heatmaps demonstrate that the network successfully ignores obstructive elements and concentrates its attention on the visible textures and morphological anomalies of the fruit. This confirms that the integration of the FasterNet and SimSPPF modules provides superior robustness in complex field environments.

However, the model presents specific limitations in low-light conditions and varying capture distances. In environments with deep shadows, some healthy coffee berries are incorrectly enclosed in black bounding boxes (Damaged category), as intense shadows mimic the dark, necrotic textures characteristic of damaged fruits [28]. Furthermore, since the training dataset primarily consisted of close-up imagery, detection accuracy slightly diminishes for distant clusters where morphological details are less defined. In these cases, the combination of shadows and reduced resolution at a distance increases the likelihood of localized misclassifications [8].

### 4.6. Performance Evaluation of M6 vs. M9

A comparative analysis between M6 and M9 showed that the M6 YOLO architecture outperformed M9 (Mean F1: 0.5758 vs. 0.3270) as reported in Table S1. The M6 revealed balanced detection [28], whereas M9 failed in Healthy (0.1538) and Damaged (0.1000). Figure 11 (further comparisons are available in Supplementary Materials Figures S1–S10 and Table S1) confirms M6’s superior spatial heatmaps versus M9’s scattered activations.

The superiority of M6 probably stems from the synergistic integration of FasterNet (FasterBlock) and SimSPPF. As demonstrated by Gao et al. [35] and Zhang et al. [36], the adoption of FasterBlocks drastically reduces redundant data flow and memory consumption. This allows the model to isolate critical pest-related textures without overloading computational resources. Furthermore, replacing the standard SPPF with SimSPPF enhances the recognition of multi-scale objects through a simplified pooling structure that streamlines local feature fusion [35], a factor essential for identifying small coffee borers in dense clusters.

Conversely, the performance decline in M9, which integrates the EMA module, could be attributed to feature redundancy and semantic misalignment within the cluttered agricultural background. While EMA is highly effective for capturing multi-scale patterns in crops [37] or maritime environments [38,39], its cross-spatial learning mechanism inadvertently prioritized background noise in our dense canopy scenario. As noted in SEF-RegNet [40], deeper models with multi-scale attention can exhibit a lack of correlation between loss and accuracy when resolutions are constrained, leading the model to “confide” in irrelevant deep features.

Our results suggest that M9’s failure to distinguish healthy berries from foliage (0.1538 F1-score) could be due to the EMA module overfitting to irregular dark patterns caused by leaf shadows—a risk of background feature learning also identified in infrared detection [39]. Unlike ECCFNet [41], which balances macro-perception and micro-focusing, the excessive complexity of M9 resulted in spatial information loss during propagation.

### 4.7. Comparative Analysis with the Literature

Recent advances in DL–based object detection have demonstrated remarkable progress in agricultural monitoring; however, substantial differences remain in terms of evaluation rigor, deployment feasibility, and interpretability. A comparative analysis with representative studies reveals both the strengths and limitations of existing approaches when contrasted with the framework proposed in this research (Table 4).

For instance, Liu et al. [42] proposed a dual module perception system combining a vision transformer-based navigation framework with a lightweight instance segmentation architecture for greenhouse robots. Their system achieved boundary F1 scores of 90.2%, 87.6%, 88.8%, and 86.3% for tomato, cucumber, pepper, and lettuce, respectively, while maintaining a navigation latency of 51.0 ms and a frame rate of 44.2 FPS under adverse conditions such as rain and fog. Despite the relevance of these results for robotic perception, the study does not report detailed detection metrics including mAP distributions, parameter counts, or computational complexity indicators such as GFLOPs. Additionally, the absence of XAI analysis and the restriction of experimental validation to greenhouse environments may limit broader applicability in open-field agricultural settings. In comparison, the methodology presented in this study explicitly integrates both efficiency metrics and interpretability verification, thereby strengthening the reliability of the detection outcomes.

Further progress in small target detection has been demonstrated by Yuan, et al. [43], who introduced the ClearSight RS framework based on YOLOv5. Their approach incorporated Dynamic Snake Convolution, a Bi-Level Routing Attention mechanism, and a high-resolution detection head to enhance boundary feature extraction and target localization. The resulting model achieved an mAP of 93.8% on the NWPU VHR 10 dataset and demonstrated strong small target discrimination on the VEDAI and DOTA datasets. Nevertheless, the evaluation focused primarily on remote sensing imagery and did not report additional operational metrics such as inference speed, parameter counts, GFLOPs, or interpretability assessments. Furthermore, the lack of real-world agricultural deployment testing limits the assessment of field level performance under dynamic environmental conditions.

Recent advances in YOLO-based detection systems have predominantly emphasized accuracy gains through architectural expansion, attention mechanisms, and hybrid modeling. For instance, Su et al. [44] integrate transformer-based segmentation with YOLOv5L6 to enhance global–local feature representation, while Dai et al. [45] incorporate Vision Transformer and CBAM modules to improve contextual reasoning under real-time constraints. Similarly, Chen et al. [46] demonstrate that multiscale fusion and attention refinements can substantially boost detection precision, albeit with reduced discriminative robustness in fine-grained scenarios. While these approaches achieve notable performance improvements, they rely on increasingly complex pipelines and are often evaluated under GPU-dependent settings, limiting their applicability to resource-constrained agricultural environments. In contrast, the present study departs from accuracy-centric design by explicitly formalizing the trade-off between detection fidelity and computational overhead. Through systematic integration of FasterNet and SimSPPF within YOLOv26 and the application of Pareto optimality, this work provides a principled framework for selecting architectures that maintain near-baseline accuracy while significantly reducing FLOPs—an aspect only marginally addressed in prior studies.

Parallel efforts targeting lightweight deployment, such as Zeng et al. [47], Liu et al. [48], Sun et al. [49], Bhat et al. [50], and Liao et al. [51], demonstrate that pruning, quantization, lightweight backbones, and knowledge distillation can substantially reduce model size and computational cost while preserving acceptable accuracy. However, these methods typically optimize post hoc compression or backbone substitution without explicitly quantifying the accuracy–efficiency frontier or validating performance under complex, real-world field conditions with dense small object distributions. Particularly, limitations persist in detecting tightly clustered or morphologically subtle targets, an issue central to coffee berry borer infestation.

**Table 4:** Summary of comparative studies employing deep learning models.

Ref	Study Aim	Model Used	Performance Metrics	Pareto
[42]	Develop a dual-module perception system integrating navigation and phenotype recognition for greenhouse robots.	Visual-transformer-based navigation; lightweight instance segmentation for phenotype detection.	Navigation: 51.0 ms delay, 44.2 FPS, jitter amplitude 1.36°; Detection boundary F1: Tomato 90.2%, Cucumber 87.6%, Pepper 88.8%, Lettuce 86.3%.	No
[43]	Improve small-target detection in remote sensing through enhanced YOLOv5 architecture.	ClearSight-RS (YOLOv5-based) with DSConv, BRA, and high-resolution detection head.	mAP 93.8% on NWPU VHR-10; highest category-wise mAP on VEDAI; strong small-target discrimination on DOTA; increased accuracy with reduced redundancy.	No
[47]	Lightweight tomato detection for mobile deployment	THYOLO (YOLOv5 + MobileNetV3 + pruning + GA optimization)	mAP 0.969; 78% params ↓; 84.15% FLOPs ↓; 26.5 FPS mobile	No
[48]	Ultra-low precision quantization for edge deployment	MPQ-YOLO (1-bit backbone, 4-bit head)	74.7% mAP (VOC), 51.5% (COCO); 16.3× complexity reduction	No
[49]	Lightweight fruit detection with improved embedded performance	G-YOLO-NK (GhostNet + distillation + feature fusion)	96.0% AP; 7.14 MB model; 11.23 FPS (Jetson Nano)	No
[50]	Edge optimization of YOLO11 via pruning and quantization	YOLO-OptiMob (YOLO11 + pruning + INT8 quantization)	Model size: 11.4 → 2.5 MB; real-time mobile inference; slight mAP drop	No
[51]	Efficient citrus detection with improved small-object performance	YOLO-MECD (YOLOv11s + EMA + CSPPC + MPDIoU)	81.6% mAP; 75.6% params ↓; improved precision & recall	No
This study	Evaluate lightweight models for coffee fruit pathology detection and identify the optimal accuracy–efficiency configuration	M0–M9 YOLO variants; M6 (FasterNet + SimSPPF)	mAP@0.5 = 0.9446, mAP@0.5:0.95 = 0.8645, Precision = 0.9264, Recall = 0.8746, Params = 2.32 M, GFLOPs = 5.12	Yes

Against this backdrop, the results obtained in the present study demonstrate a balanced combination of detection accuracy, computational efficiency, and interpretability. Experimental evaluation revealed that the baseline model M0 achieved the highest detection accuracy with an mAP@0.5 of 0.9534. However, the integration of FasterNet and SimSPPF modules in model M6 produced the most favorable trade–off between accuracy and computational efficiency, achieving an mAP@0.5 of 0.9446 with only 5.12 GFLOPs and 2.32 million parameters. Pareto efficiency analysis further confirmed M6 as the optimal configuration among all tested architectures, indicating superior efficiency performance relative to the evaluated model variants. Importantly, Eigen CAM based XAI validation demonstrated that the model consistently focused on symptomatic regions of coffee fruits even under partial occlusion, thereby confirming the reliability of the learned visual representations.

Although occasional performance degradation was observed under low illumination conditions and long–distance image capture, where shadows and reduced spatial resolution occasionally produced misclassification, the proposed framework nevertheless demonstrates strong robustness for real world phytosanitary monitoring. Collectively, these findings indicate that the approach presented in this study advances the current state of the art by combining efficient lightweight detection, interpretability validation, and edge compatible deployment for the monitoring of *H. hampei* damage in clustered fruits of *Coffea arabica*.

### 4.8. Limitations

Despite the promising results obtained, several limitations should be acknowledged. First, although the proposed model demonstrated strong detection accuracy and computational efficiency, performance degradation was observed under challenging imaging conditions, particularly in low illumination environments and long–distance captures where shadows and reduced spatial resolution occasionally resulted in misclassification. Second, the dataset primarily consisted of images captured under specific field conditions and geographic contexts, which may limit the generalizability of the model across diverse coffee production systems with different canopy structures, lighting variability, and fruit clustering patterns. Third, the operational reliability of the wireless sensor network is constrained by the physical characteristics of the agricultural environment. Following the findings of Lloret et al. [22] regarding signal propagation in rural areas, the dense foliage of coffee trees introduces significant vegetation loss, estimated at approximately 1.2 dB/m for 802.11 g signals. This factor limits the effective WiFi communication range to approximately 30–34 m, necessitating a high density of Fog Devices or the implementation of complex multi-hop routing to maintain stable connectivity for high-definition streaming. Consequently, the scalability of the system in topographically irregular or extremely dense plantations remains a challenge that requires further field characterization. Fourth, further evaluation focused on the deployment of the model on a single low-cost edge computing platform, namely the Raspberry Pi 5, would be needed. Therefore, additional benchmarking on other embedded systems and mobile agricultural robotics platforms would provide a more comprehensive assessment of real-world operational scalability.

Furthermore, while Eigen CAM based XAI analysis confirmed that the model concentrated on symptomatic fruit regions, deeper interpretability analyses integrating multiple explainability frameworks could further strengthen model transparency and reliability. Addressing these limitations through larger multi regional datasets, expanded edge device validation, and more extensive interpretability assessments will be essential to enhance the robustness and practical adoption of automated detection systems for the monitoring of damage caused by the CBB in *Coffea arabica* cultivation systems.

## 5. Conclusions and Future Prospectives

This study demonstrates that the integration of lightweight architectural enhancements can substantially improve the balance between detection accuracy and computational efficiency in coffee fruit pathology detection systems. In particular, the combined incorporation of FasterNet and SimSPPF modules significantly enhanced the overall performance of the evaluated models. Among the tested configurations, Model M6 exhibited the most favorable accuracy–efficiency trade off, achieving a high detection accuracy with mAP@0.5 of 0.9446 while maintaining a reduced computational complexity of only 5.12 GFLOPs. Pareto efficiency analysis further confirmed the superiority of this configuration over both the baseline architecture and other module variants evaluated in this work. In addition, Eigen CAM–based XAI analysis verified that the model consistently concentrated on pathology-relevant fruit regions, even in scenarios characterized by partial occlusion. These findings indicate that the proposed framework is capable of learning reliable visual representations for the detection of damage caused by the CBB in fruits of *Coffea arabica*. Nevertheless, a decline in classification confidence was observed in low illumination environments and long distance capture conditions, where shadow interference and reduced image resolution occasionally affected detection reliability.

Future research should therefore focus on improving the robustness and generalization capacity of the proposed system under diverse real-world conditions. Expanding the dataset to incorporate a broader range of illumination levels, acquisition distances, and field environments would significantly enhance model adaptability to the variability encountered in commercial plantations. Moreover, the integration of adaptive illumination correction mechanisms, distance-aware feature-enhancement strategies, and self-supervised pretraining approaches may further mitigate the limitations associated with shadow dominated scenes and low contrast imagery. Another promising direction involves the incorporation of multimodal sensing frameworks that combine conventional RGB imaging with complementary modalities such as near infrared or thermal sensing, thereby improving the discrimination between genuine necrotic tissue patterns and shadow-induced visual artifacts. Finally, the development of an integrated edge fog cloud computational pipeline could enable continuous real-time monitoring within precision agriculture systems. Such an architecture would facilitate scalable in-field decision support for farmers and agronomists, ultimately contributing to a more efficient and sustainable management of infestations caused by *H. hampei* in coffee production systems.

## Figures and Tables

**Figure 11:**
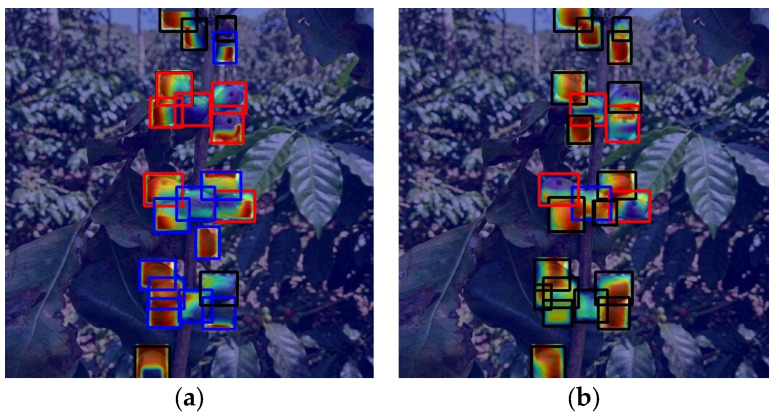
Comparative Eigen-CAM interpretability analysis: (**a**) M6 architecture vs. (**b**) M9 architecture.

**Table 1:** Mathematical formulation for model efficiency metrics and selection criteria.

Parameter	Mathematical Formula	
LPS	$mAP@0.5/T_{inf}$	(1)
GESI	mAP@0.5/GFLOPs	(2)
PEI	$mAP@0.5/(P\times 10^{6})$	(3)
Custom Score	$\left(F{1-Score}_{norm}+LPS_{norm}+PEI_{norm}+GESI_{norm}\right)/4$	(4)
Pareto Frontier Analysis	mAP@0.5(M)≥mAP@0.5(M)∩GFLOPs(M)≤GFLOPs(M)	(5)

where $T_{inf}$ is the inference time in ms, P is the number of parameters, and M is the model.

**Table 2:** Performance metrics and computational complexity analysis for different YOLO-based model configurations.

Model	FasterNet	SimSPPF	EMA	mAP@0.5	mAP@0.5:0.95	Precision	Recall	Parameters	GFLOPs
M0				0.9534	0.8735	0.9179	0.9028	3,006,233	8.087
M1				0.9466	0.8667	0.9356	0.8659	2,582,737	6.315
M2				0.9426	0.8629	0.9086	0.9005	2,375,421	5.192
M3	✓			0.9402	0.8593	0.923	0.8583	2,319,101	5.124
M4		✓		0.9357	0.8544	0.9402	0.8587	2,375,421	5.192
M5			✓	0.9298	0.8489	0.9273	0.8234	2,376,093	5.258
M6	✓	✓		0.9446	0.8645	0.9264	0.8746	2,319,101	5.124
M7		✓	✓	0.9272	0.8455	0.9225	0.8571	2,376,093	5.258
M8	✓		✓	0.921	0.834	0.8999	0.832	2,319,773	5.19
M9	✓	✓	✓	0.9142	0.8306	0.8753	0.8503	2,319,773	5.19

M0 represents the YOLOv8n baseline; M1 denotes the YOLOv11n baseline, and M2 is the YOLOv26n baseline. M3 to M9 are the proposed YOLOv26n architectural variants. FasterNet integrates PConv for memory efficiency; SimSPPF refers to the Simplified Spatial Pyramid Pooling–Fast module; EMA indicates the integration of the Efficient Multi-Scale Attention mechanism.

**Table 3:** Multi-objective ranking and Pareto efficiency results for the evaluated coffee fruit detection models.

Model	Acquired Score	Pareto Frontier
M2	0.915	No
M3	0.882	No
M4	0.864	No
M6	0.855	Yes
M7	0.775	No
M5	0.674	No
M9	0.653	No
M8	0.616	No
M1	0.614	Yes
M0	0.250	Yes

## Data Availability

The data provided can be found within the article. The original contributions made in this study are included in the document; any additional inquiries can be directed to the author or authors responsible.

## Supplementary Materials

The following supporting information can be downloaded at: https://www.mdpi.com/article/10.3390/s26072212/s1, Figure S1. XAI-based interpretability analysis of coffee berry pathology detection using Eigen-CAM for M0. Figure S2. XAI-based interpretability analysis of coffee berry pathology detection using Eigen-CAM for M1. Figure S3. XAI-based interpretability analysis of coffee berry pathology detection using Eigen-CAM for M2. Figure S4. XAI-based interpretability analysis of coffee berry pathology detection using Eigen-CAM for M3. Figure S5. XAI-based interpretability analysis of coffee berry pathology detection using Eigen-CAM for M4. Figure S6. XAI-based interpretability analysis of coffee berry pathology detection using Eigen-CAM for M5. Figure S7. XAI-based interpretability analysis of coffee berry pathology detection using Eigen-CAM for M6. Figure S8. XAI-based interpretability analysis of coffee berry pathology detection using Eigen-CAM for M7. Figure S9. XAI-based interpretability analysis of coffee berry pathology detection using Eigen-CAM for M8. Figure S10. XAI-based interpretability analysis of coffee berry pathology detection using Eigen-CAM for M9. Table S1. Detailed F1-score breakdown for the CBB detection sample analyzed in the XAI study for the Figures S1–S10.

## Abbreviations

The following abbreviations are used in this manuscript:

CBB	Coffee Berry Borer
YOLO	You Only Look Once
DL	Deep Learning
AI	Artificial Intelligence
CNN	Convolutional Neural Network
XAI	Explainable Artificial Intelligence
mAP	Mean Average Precision
GFLOPs	Giga Floating-Point Operations per Second
SPPF	Spatial Pyramid Pooling–Fast
EMA	Exponential Moving Average
CAM	Class Activation Map
ROI	Region of Interest
IoU	Intersection over Union
FPS	Frames Per Second
WSN	Wireless Sensor Network
RGB	Red–Green–Blue (Color Model)
