# Supplementary material for: Proposal for Computationally Efficient Fog Computing System for Coffee Berry Borer Detection via Optimized YOLOv26
Source: Sensors (Basel). 2026 Apr 3;26(7):2212. doi: 10.3390/s26072212 (PMC13075308; doi:10.3390/s26072212)
Supplement: Supplementary file 1 [file sensors-26-02212-s001.zip › sensors-4224227-supplementary.pdf]

Computationally Efficient Fog Computing System for Real–Time Coffee Berry Borer Detection via Optimized YOLOv26

Ingrid P. Huaman-Pacco [1], Erwin J. Sacoto-Cabrera [2], Vinie Lee Silva-Alvarado [3], Ali Ahmad [3], Sandra Sendra [3], Jaime Lloret [3] and Edison Moreno-Cardenas [1,4,]

1 TESLA Laboratory, Universidad Nacional de San Antonio Abad del Cusco, Cusco 08003, Peru; 182972@unsaac.edu.pe
2 GIHP4C, Universidad Politécnica Salesiana, Cuenca 010102, Ecuador; esacoto@ups.edu.ec
3 Instituto de Investigación para la Gestión Integrada de Zonas Costeras, Universitat Politécnica de València, C/Paranimf, 1, Grao de Gandia 46730, Valencia, Spain; vlsilalv@doctor.upv.es, aahmad1@upv.es, sansenco@upv.es, jlloret@dcom.upv.es
4 Technology and Engineering Group, EM Research & Tech, 08003 Cusco, Perú; edison.moreno@emresearchtech.com

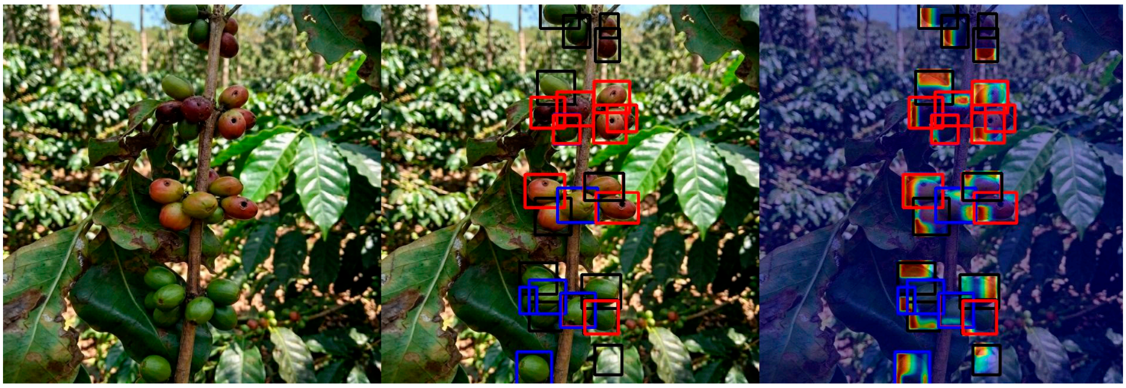

Figure S1. XAI-based interpretability analysis of coffee berry pathology detection using Eigen-CAM for M0.

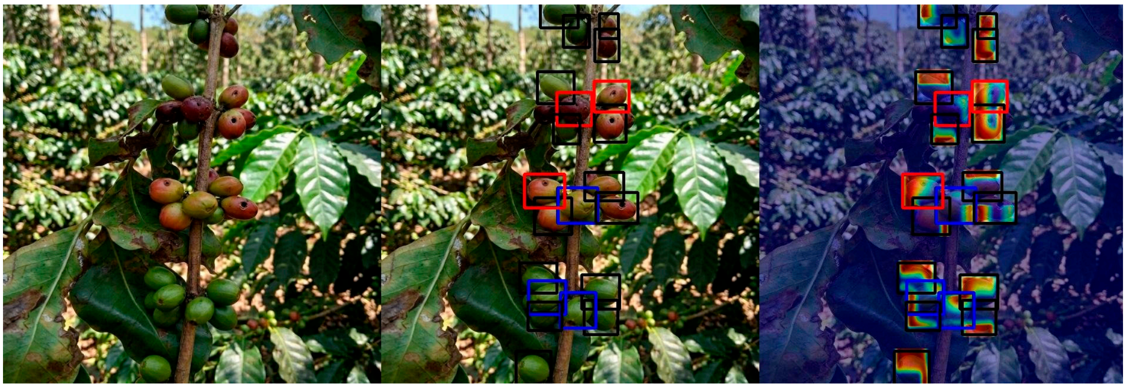

Figure S2. XAI-based interpretability analysis of coffee berry pathology detection using Eigen-CAM for M1.

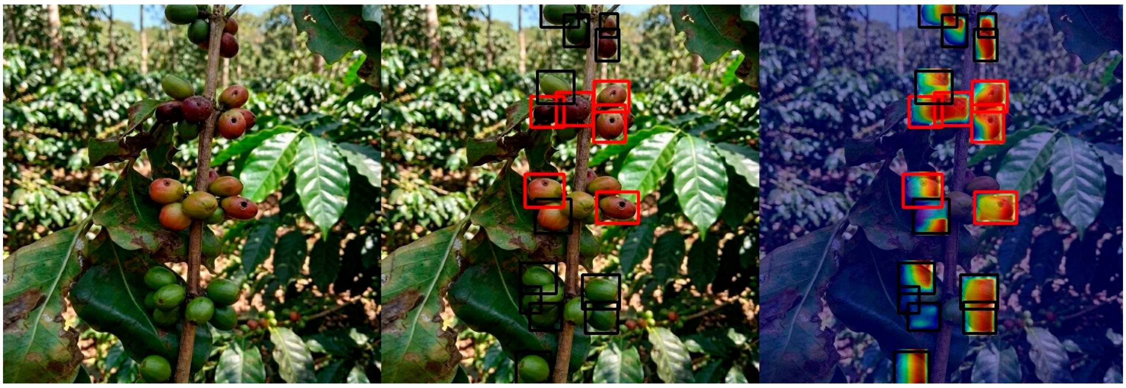

Figure S3. XAI-based interpretability analysis of coffee berry pathology detection using Eigen-CAM for M2.

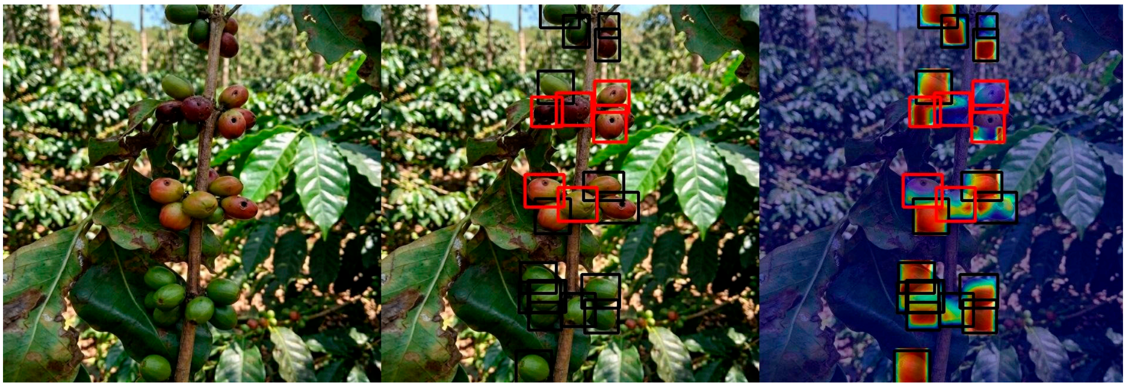

Figure S4. XAI-based interpretability analysis of coffee berry pathology detection using Eigen-CAM for M3.

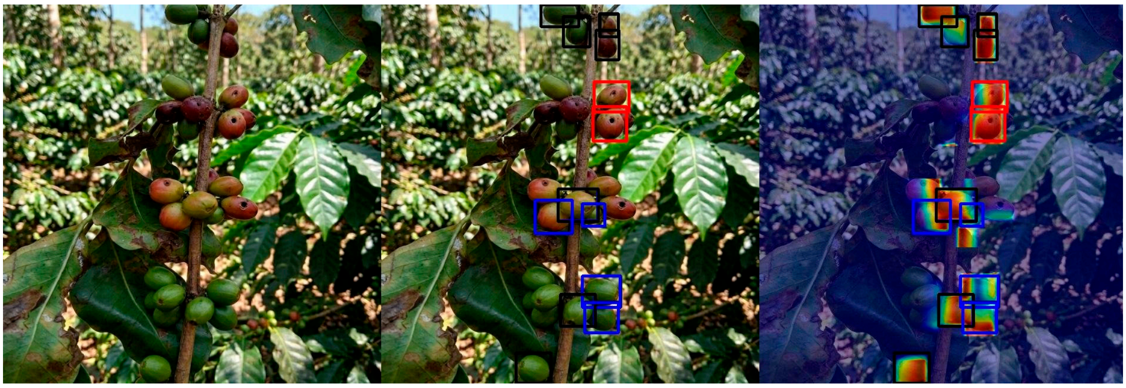

Figure S5. XAI-based interpretability analysis of coffee berry pathology detection using Eigen-CAM for M4.

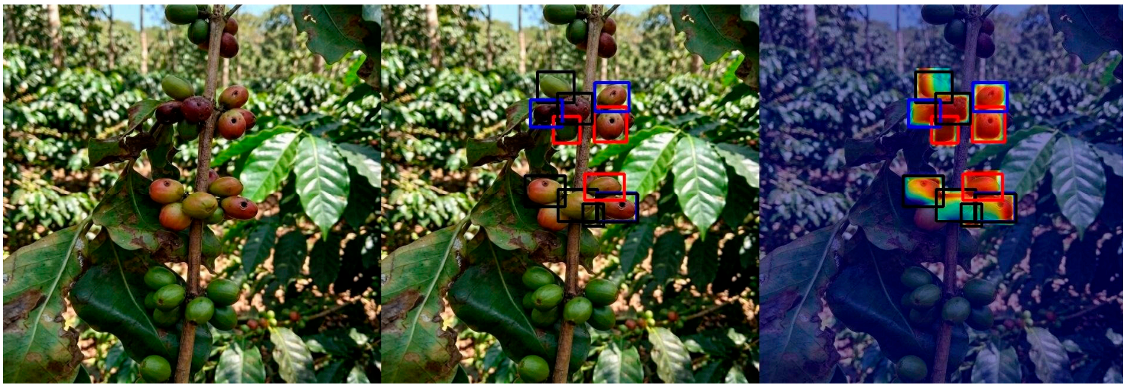

Figure S6. XAI-based interpretability analysis of coffee berry pathology detection using Eigen-CAM for M5.

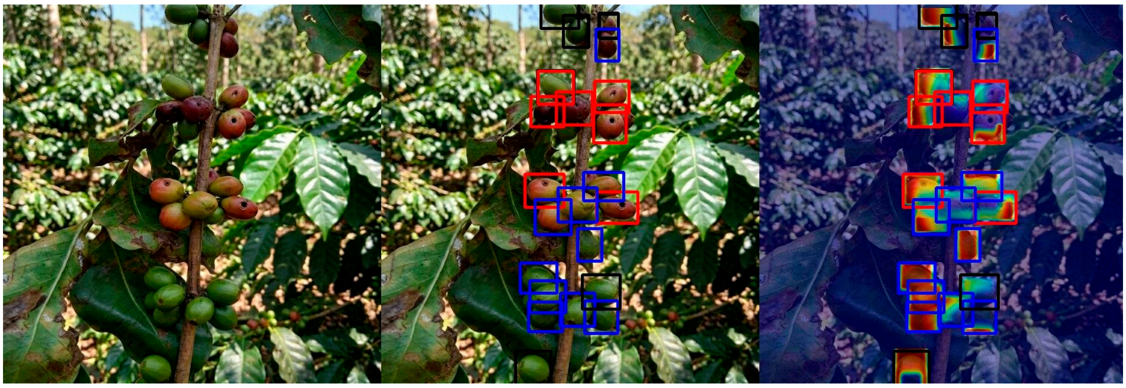

Figure S7. XAI-based interpretability analysis of coffee berry pathology detection using Eigen-CAM for M6.

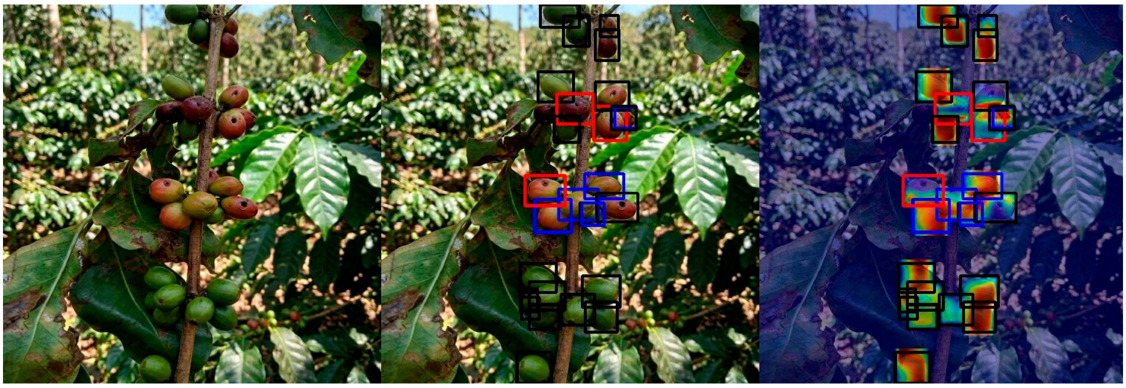

Figure S8. XAI-based interpretability analysis of coffee berry pathology detection using Eigen-CAM for M7.

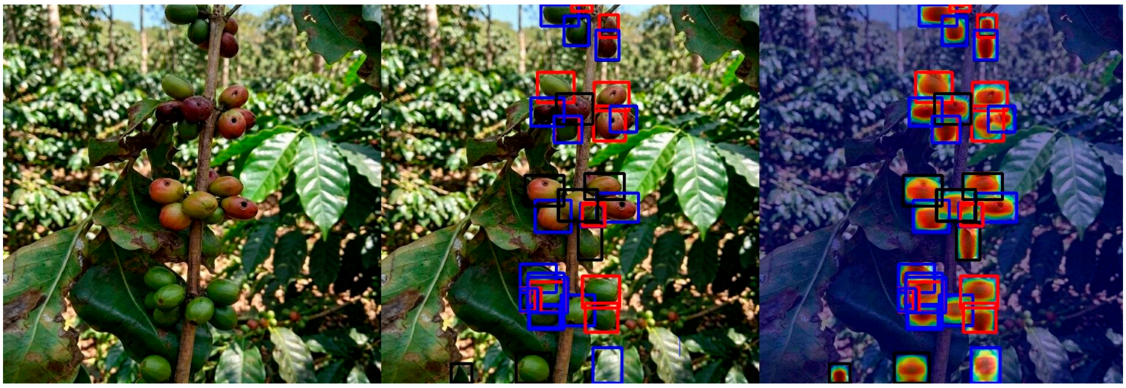

Figure S9. XAI-based interpretability analysis of coffee berry pathology detection using Eigen-CAM for M8.

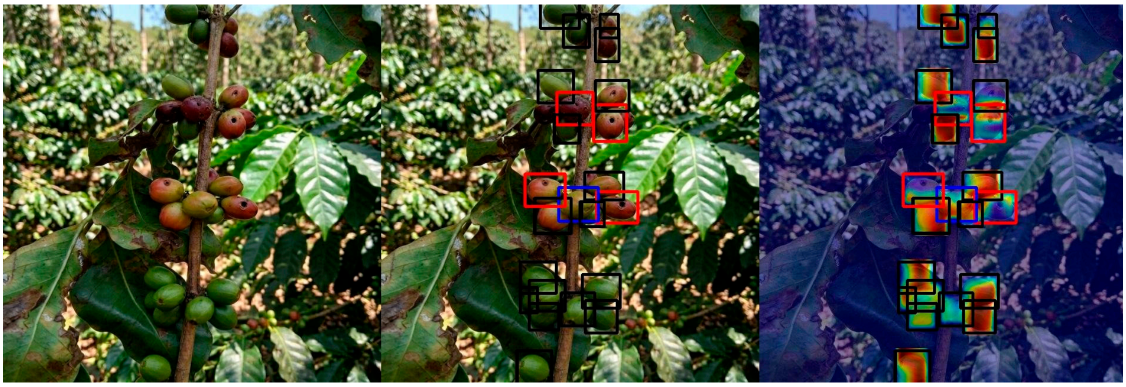

Figure S10. XAI-based interpretability analysis of coffee berry pathology detection using Eigen-CAM for M9.

Table S1. Detailed F1-score breakdown for the CBB detection sample analyzed in the XAI study for the Figures S1-10.

**Model**	**F1-score**			
	**Healthy**	**Infected**	**Damaged**	**Mean**
**M0**	**0.4706**	**0.7143**	**0.1538**	**0.4462**
**M1**	**0.4000**	**0.6000**	**0.1250**	**0.3750**
M2	0.0000	0.7692	0.1429	0.3040
M3	0.0000	0.7143	0.1250	0.2798
M4	0.2500	0.4444	0.0000	0.2315
M5	0.0000	0.2000	0.0000	0.0667
**M6**	**0.7273**	**0.7143**	**0.2857**	**0.5758**
M7	0.3529	0.6000	0.0952	0.3494
M8	0.5600	0.3529	0.0000	0.3043
M9	0.1538	0.7273	0.1000	0.3270
